# High Performance Liquid Chromatographic Assay for the Simultaneous Determination of Posaconazole and Vincristine in Rat Plasma

**DOI:** 10.1155/2015/743915

**Published:** 2015-12-22

**Authors:** Hadeel A. Khalil, Ahmed F. El-Yazbi, Tarek S. Belal, Dalia A. Hamdy

**Affiliations:** ^1^Pharmaceutical Analytical Chemistry Department, Faculty of Pharmacy, Alexandria University, Alexandria 21521, Egypt; ^2^Department of Pharmacology, Faculty of Pharmacy, Alexandria University, Alexandria 21521, Egypt

## Abstract

*Purpose*. Developing a validated HPLC-DAD method for simultaneous determination of posaconazole (PSZ) and vincristine (VCR) in rat plasma.* Methods*. PSZ, VCR, and itraconazole (ITZ) were extracted from 200 *μ*L plasma using diethyl ether in the presence of 0.1 M sodium hydroxide solution. The organic layer was evaporated in vacuo and dried residue was reconstituted and injected through HC-C18 (4.6 × 250 mm, 5 *μ*m) column. In the mobile phase, acetonitrile and 0.015 M potassium dihydrogen orthophosphate (30 : 70 to 80 : 20, linear gradient over 7 minutes) pumped at 1.5 mL/min. VCR and PSZ were measured at 220 and 262 nm, respectively. Two Sprague Dawley rats were orally dosed PSZ followed by iv dosing of VCR and serial blood sampling was performed.* Results*. VCR, PSZ, and ITZ were successfully separated within 11 min. Calibration curves were linear over the range of 50–5000 ng/mL for both drugs. The CV% and % error of the mean were ≤18% and limit of quantitation was 50 ng/mL for both drugs. Rat plasma concentrations of PSZ and VCR were simultaneously measured up to 72 h and their calculated pharmacokinetics parameters were comparable to the literature.* Conclusion.* The assay was validated as per ICH guidelines and is appropriate for pharmacokinetics drug-drug interaction studies.

## 1. Introduction

Vincristine (VCR) is a mitotic inhibitor antineoplastic agent that is used mainly in combination chemotherapy regimens for acute and chronic leukemia, lymphomas, including Hodgkin's disease and non-Hodgkin's lymphomas, and multiple myeloma [1]. Posaconazole (PSZ) is an oral triazole antifungal used in the treatment of severe oropharyngeal candidiasis, invasive aspergillosis, and other fungal infections in patients who are resistant to, or intolerant of, other antifungal drugs. PSZ is also given for prophylaxis in patients who are at high risk for invasive fungal disease due to immunosuppression, such as haematopoietic stem cell transplant recipients with graft-versus-host disease, or those with haematological malignancies with prolonged neutropenia as a result of chemotherapy [1, 2]. The coadministration of azoles (as prophylaxis or treatment of fungal infections) and VCR has been shown to increase VCR neurotoxic effects due to the inhibition of cytochrome P450 (CYP) isoform 3A4, for which VCR is a substrate [2]. Those neurotoxic symptoms usually appear as constipation and peripheral neurotoxicity [3]. In addition, few case reports have illustrated the possible exacerbation of VCR toxicity by coadministration of PSZ in children and young adults where fluctuations in level of consciousness and seizures have been observed [4].

PSZ has been determined in human plasma samples using capillary electrophoresis [5], HPLC with fluorescence detection [6], and HPLC-tandem mass spectrometry (HPLC-MS-MS) methods [7, 8]. Additionally, the simultaneous determination of azole antifungals including PSZ in plasma/serum samples gained a growing interest in the past few years. Different chromatographic methods have been described for this task such as micellar electrokinetic chromatography (MEKC) [9] and several liquid chromatographic methods coupled with tandem mass spectrometry [10, 11], fluorescence detection [12], and UV detection [13–16]. On the other hand, estimation of VCR in plasma samples has been carried out using HPLC-UV detection [17, 18], UPLC-MS-MS [19], and several HPLC-MS-MS methods [20–23]. Besides, an HPLC-MS-MS method was presented for the simultaneous determination of VCR and actinomycin-D in human dried blood spots [24]. In addition, quantification of intracellular VCR concentrations in children with acute lymphoblastic leukemia has been described using HPLC with electrochemical detection procedure [25]. Moreover, capillary zone electrophoresis coupled with electrochemical detection has been employed to study the uptake kinetics of VCR by human erythrocytes [26]. Finally, pharmacokinetics of PSZ and VCR in rats have been separately discussed in only two published reports [27, 28].

Although PSZ-VCR drug-drug interaction has been exposed in several previous reports, the concurrent determination of PSZ and VCR has not been tackled yet since no analytical reports can be found in the literature. In this report, we describe the first simple and reliable RP-HPLC with diode array detection procedure for the simultaneous determination of PSZ and VCR in rat plasma. The structurally related azole antifungal drug (itraconazole, ITZ) has been used as an internal standard. The proposed method has been applied in a preliminary pharmacokinetics drug interaction study in rats. An analytical method capable of simultaneously measuring two drugs would be a valuable tool, facilitating the measurement of pharmacokinetics using a single blood draw for each time point with no need to split the sample for processing through two assay methods. In serial blood collection studies involving rats, in particular, this is an important consideration due to the need to keep cumulative blood volume withdrawal to a minimum.

## 2. Experimental

### 2.1. Materials and Reagents

Posaconazole and vincristine sulfate powders (purity >99% for both) were purchased from Selleckchem (Houston, TX, USA). Itraconazole was a kind gift from Nifty Labs Pvt. Ltd., Hyderabad, India. HPLC grade methanol and acetonitrile (Fisher Scientific UK Limited, Loughborough, Leicestershire, UK), analytical grade potassium dihydrogen orthophosphate (Riedel-de-Haën, Germany), and high purity distilled water were used. Noxafil oral suspension labeled to contain 40 mg/mL PSZ (Patheon Inc., Ontario, Canada,) was purchased from Schering-Plough S.A. Vincarine vials labeled to contain 1 mg/mL vincristine sulfate solution (EIMC United Pharmaceuticals, Badr city, Cairo, Egypt) were obtained from the local market.

### 2.2. Chromatographic Conditions

The HPLC-DAD system consisted of Agilent 1200 series (autoinjector, quaternary pump, vacuum degasser and diode array, and multiple wavelength detectors G1315 C/D and G1365 C/D) connected to a computer loaded with Agilent ChemStation Software (Agilent Technologies, Santa Clara, CA, USA). The DAD wavelength was set at 220 and 262 nm. The chromatographic separation was achieved using a HC-C18 (4.6 × 250 mm, 5 *μ*m particle size) column attached to HC-C18 (4.6 × 12.5 mm, 5 *μ*m particle size) guard column (Agilent Technologies, Santa Clara, CA, USA). The gradient elution composed the mobile phase: acetonitrile and 0.015 M potassium dihydrogen orthophosphate (30 : 70 to 80 : 20, linear over 7 minutes) pumped at 1.5 mL/min. Injection volume was 80 *μ*L. All determinations were performed at 25°C.

### 2.3. Stock and Standard Solutions

Stock solutions of PSZ and VCR sulphate (100 mg/L) were separately prepared in methanol. A 10 mg/L stock solution of the internal standard (ITZ) was prepared in methanol. To prepare samples for the calibration curves and validation assessment, three working standard solutions (10, 1, and 0.1 mg/L) of PSZ and VCR were prepared freshly by successive 1/10 dilutions of the stock solutions with methanol.

### 2.4. Extraction Procedure

The ITZ (0.03 mL) was added to each 0.2 mL rat plasma sample in a glass test tube. To the rat plasma sample, 0.03 mL of 0.1 M NaOH and 6 mL of diethyl ether were added. Because methanol was present in the standard curve samples, an equivalent amount was added to the test samples as well (0.2 mL). The tubes were covered, vortex-mixed for 2 min at high speed, and then subsequently centrifuged for 10 min at ~2500 ×g. The organic layer was transferred to new glass tubes and evaporated to dryness* in vacuo* (Christ rotational vacuum concentrator, Germany). The tubes were placed in a −20°C freezer until analysis time. The residues were reconstituted in 100 *μ*L methanol of which 80 *μ*L volumes were injected into the HPLC.

### 2.5. Recovery

The plasma recoveries were determined for PSZ, VCR, and ITZ at concentration level 2500 ng/mL in rat plasma using four replicates for each concentration. The extraction efficiency was determined by comparing the peak areas of each analyte to the peak areas of the same amounts directly injected to the instrument without extraction.

### 2.6. Validation

Calibration curves were constructed using samples of 0.2 mL rat plasma containing PSZ, VCR, and IS. The curve ranged from 50 to 5000 ng/mL for both PSZ and VCR. The ratios of PSZ and VCR peak areas to IS peak area were calculated and plotted versus the expected PSZ or VCR concentrations. Owing to the wide range of concentrations, the calibration curve data were weighed by a factor of 1/*x* ^2^ for both drugs. Intraday accuracy and precision of the assay were determined using four sample replicates of 50, 500, and 2500 ng/mL for each of PSZ and VCR in rat plasma in the same day. The same concentrations were analyzed in three separate days for assessment of the interday accuracy and precision. For each daily run, concentrations were determined by comparison with a calibration curve prepared simultaneously on the same day of analysis. Precision was determined using percentage coefficient of variation (CV%) and bias was assessed using percentage error of the mean.

### 2.7. Application to a Drug Interaction PK Study

To evaluate the applicability of this method in vivo, two rats (200–250 g) were given 40 mg/kg PSZ orally followed by 0.1 mg/kg VCR i.v. through the tail vein after 30 min of oral dosing. The protocol was approved by the Ethics Committee, Faculty of Pharmacy, Alexandria University. On the day before the pharmacokinetic study, food was withheld overnight. On the next morning, animals were transferred to metabolic cages to conduct the pharmacokinetic experiments. Serial blood samples were collected at 0.50, 0.75, 0.92, 1.33, 2.0, 3.5, 6.0, 8.0, 24.0, 48.0, and 72.0 h after oral PSZ dose using retroorbital sampling. Plasma was separated by centrifugation of the blood at ~2500 ×g for 3 min. The samples were kept at −20°C until analysis time.

### 2.8. Data and Statistical Analysis

Noncompartmental methods were applied to calculate the pharmacokinetic parameters. The elimination rate constant (*λz*) was calculated by subjecting the plasma concentrations in the terminal phase to linear regression analysis. *t*(1/2) was calculated using the equation *t*(1/2) = 0.693/*λz*. AUC_0–*∞*_ was calculated using the combined log-linear trapezoidal rule from time 0 h after dose to the time of the last measured concentration, plus the quotient of the last measured concentration divided by *λz*. The concentration at time 0 h after i.v. dosing (*C*_o_) was estimated by back extrapolation of the log-linear regression line using the first three measured plasma concentrations to time 0. The clearance was calculated as the quotient of dose to AUC_0–*∞*_. All compiled data were reported as mean ± SD unless otherwise indicated.

## 3. Results

**Figure 1:**
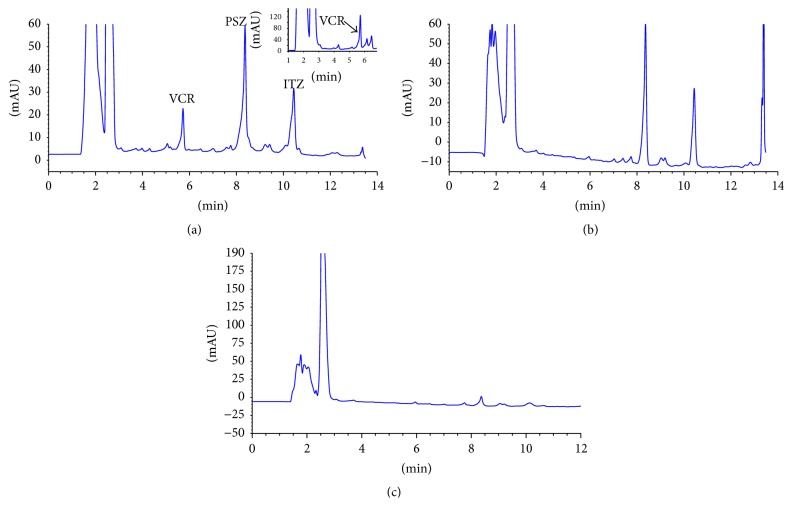
Chromatogram of (a) rat plasma spiked with 2500 ng/mL of VCR and PSZ, (b) rat plasma obtained after 6 h of oral PSZ dosing, and (c) blank rat plasma measured at 262 nm. The inset shows a section of a chromatogram for rat plasma spiked with 2500 ng/mL of VCR measured at 220 nm.

VCR, PSZ, and ITZ eluted at retention times 5.9, 8.3, and 10.4 min, respectively (Figure 1). The total analysis run time was ~11 min. The three peaks were almost symmetrical with excellent baseline separation and no interferences from endogenous substances in plasma. The column separation factor (*α*) and resolution factor for VCR and PSZ were calculated to be 1.58 and 14.5 for VCR and 1.32 and 7.95 for PSZ, respectively. The column capacity factors (*K*′) for VCR, PSZ, and ITZ were calculated to be 2.43, 3.83, and 5.05, respectively.

**Figure 2:**
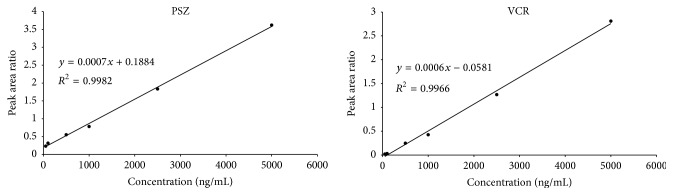
Linearity and regression for the determination of PSZ and VCR in rat plasma.

The VCR extraction efficiency recovery was found to be 75.7 ± 5.4% while PSZ and ITZ showed 103.9 ± 3.1% and 103.6 ± 5.4% (~100%) recovery, respectively. The assay linearity for VCR and PSZ calculated by measuring the peak area ratios of analyte : internal standard within the concentration range of 50 to 5000 ng/mL showed high linearity with a mean *r* ^2^ ≥ 0.997 for both drugs (Figure 2). The mean weighted slope and intercept were found to be 0.0007 ± 5.09 × 10^−5^ and 0.315 ± 0.375 for PSZ, respectively, and 0.0004 ± 0.0002 and −0.002 ± 0.0037 for VCR, respectively.

The validation data showed the assay to be sensitive, accurate, and precise, with the intraday and interday CV% values less than or equal to 13.2% and 8.78% for both drugs, respectively (Table 1). The mean interday error was less than 13.1% for both drugs. Since both CV% of interday and intraday assessment and interday mean error yielded values less than 20% at the lowest concentration tested, the lower limit of quantitation (LLQ) based on 0.2 mL of rat plasma was 50 ng/mL for both drugs.

**Figure 3:**
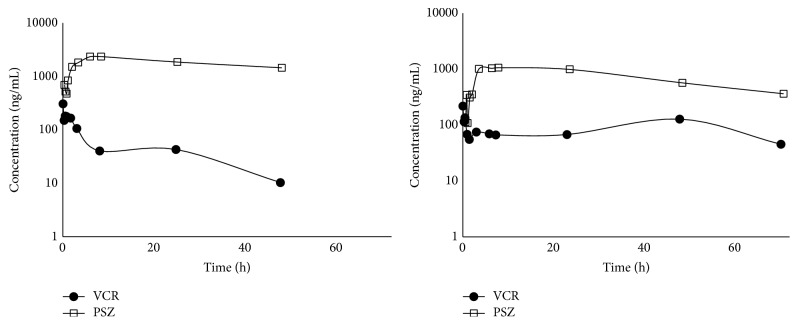
Plasma concentration versus time curves for PSZ and VCR in two rats that were given 40 mg/kg PSZ orally followed by i.v. dosing of 0.1 mg/kg VCR 30 minutes later.

In both rats dosed with 40 mg/Kg PSZ orally followed by 0.1 mg/Kg VCR i.v. 0.5 h later, the PSZ showed higher plasma concentrations than VCR at all time points (Figure 3). The PSZ Cmax for the two rats were 2344 and 1056 ng/mL and the VCR *C*_o_ were 376 and 228 ng/mL, respectively. The PSZ AUC_0–*∞*_ were 206459 and 67744 ng·h/mL, whereas the VCR AUC_0–*∞*_ were 2347 and 2568 ng·h/mL for rat 1 and rat 2, respectively. The PSZ tmax was found to be 8.4 and 7.8 h for both rats 1 and 2, respectively. The VCR clearance and half-life were calculated to be 0.043 L/h/Kg and 19.2 h for rat 1 and 0.038 L/h/Kg and 14.8 h for rat 2, respectively. The assay managed to measure the plasma concentrations for both drugs up to 72 h after dose.

## 4. Discussion

This paper demonstrates an easy, rapid, sensitive HPLC assay for the simultaneous determination of VCR and PSZ in rat plasma. It successfully measures their plasma concentrations and calculates their PK parameters up to 72 h after dose. It is worth mentioning that the assay was applied by 3 different analysts on two different HPLC apparatus and yielded similar results with minor changes in retention times and total runtime. The assay was also successfully applied to determine both drugs' concentrations in other matrices, rat's liver tissue homogenate, hyperlipidemic rat's plasma, and liver tissue homogenate, in addition to Sorenson buffer (pH = 7.4), with excellent linearity and same limit of quantification (data not shown).

During assay optimization, the authors utilized the exact chromatographic conditions that they recently published for the HPLC determination of PSZ in bulk form and suspension dosage form [29]. However, HC-C18 instead of Zorbax SB-C18 column was utilized and HC-C18 guard column was added. Despite the fact that VCR was detected at 262 nm (Figure 1), it showed better sensitivity and yield at the short wavelength 220 nm. This demonstrates the importance of the multiple wavelength detector (DAD) which offers the advantage of measuring each analyte at its optimum wavelength, thus improving sensitivity.

During the optimization of the extraction procedures, the authors tried the direct protein precipitation method and liquid extraction using diethyl ether, ethyl acetate, or methylene chloride. Both direct protein precipitation and diethyl ether extraction procedures showed similar recoveries. However, the extraction procedure was preferred to protect the column materials and for the ease of the evaporation step* in vacuo* compared to the direct protein precipitation one. Solid phase extraction was not attempted due to its relatively higher cost when working with large number of samples within the future planned PK studies.

The PSZ and VCR retention times using our simultaneous assay lie within the reported retention times for PSZ and VCR, respectively [13–18]. There is a slight interference from an endogenous plasma peak in the PSZ peak; this interference resulted in the fact that we could not quantify the PSZ peak accurately below the 50 ng/mL concentration; however, as mentioned before, it did not interfere with any of the validation parameters within our concentration range 50–5000 ng/mL.

The pharmacokinetics parameters reported for PSZ and VCR in rat were compared to the only two similar manuscripts found in literature [30, 31]. The VCR clearance came within the lower border of that reporting 0.12 ± 0.08 L/h/Kg and showed similar terminal phase half-life to that reporting 14.3 ± 6.3 h [30]. Similarly, PSZ mean AUC and *C*_max_ were close to those reported previously [31]. The slight variability could be due to the concomitant administration with VCR or difference between our SD rats and the Crl : CD BR reported [32]. It is worth mentioning that, for the future planned elaborated VCR-PSZ drug interaction study, the design of the drug interaction needs to be modified to mimic the actual conditions occurring in humans. However, for the purpose of proving the appropriateness of the analytical technique to study such interaction, we successfully dosed only a single dose of each drug and managed to measure it using our DAD detector for up to 72 h which is advantageous over similar HPLC-DAD assays reported for each drug alone.

## 5. Conclusion

This paper describes the first validated method for the simultaneous determination of posaconazole and vincristine in rat plasma. The proposed HPLC-DAD method has been applied in the investigation of their pharmacokinetics in rats up to 72 h after dose. The obtained pharmacokinetics parameters for posaconazole and vincristine are in good agreement with those reported in previous reports. The developed method is simple, sensitive, and suitable for the estimation of both drugs in comprehensive drug-drug interaction pharmacokinetic studies in rats.

## Supplementary Material

Posaconazole and itraconazole were measured at 262 nm while vincristine showed better sensitivity when measured at 220 nm. The figures below illustrate the vincristine, posaconazole and itraconazole peaks when measured at 220 nm.

## Figures and Tables

**Table 1:** Precision and accuracy for the determination of PSZ and VCR in rat plasma using the proposed HPLC-DAD method.

Nominal concentration ng/mL	Drug	Intraday mean ± SD (intraday CV%)			Interday mean ± SD, ng/mL	Interday CV%	Interday mean error%
50	PSZ	57.3 ± 10.5 (18.2)	54.9 ± 9.54 (17.4)	57.4 ± 0.86 (1.49)	56.5 ± 0.05	1.36	13.1
	VCR	50.3 ± 5.99 (11.9)	56.2 ± 2.21 (3.93)	47.3 ± 3.07 (6.48)	51.3 ± 4.5	8.78	2.53

500	PSZ	539 ± 29.8 (5.54)	516 ± 68.3 (13.2)	520 ± 32.1 (6.18)	524 ± 12.0	2.28	4.99
	VCR	528 ± 27.5 (5.21)	495 ± 17.4 (3.50)	540 ± 56.9 (10.5)	521 ± 23.0	4.41	4.25

2500	PSZ	2460 ± 383 (15.6)	2413 ± 127 (5.25)	2579 ± 270 (10.5)	2483 ± 85.7	3.45	−0.65
	VCR	2494 ± 282 (11.3)	2646 ± 186 (7.03)	2476 ± 264 (10.7)	2539 ± 93.6	3.69	1.54
