# Supplementary material for: High Performance Liquid Chromatographic Assay for the Simultaneous Determination of Posaconazole and Vincristine in Rat Plasma
Source: Int J Anal Chem. 2015 Dec 22;2015:743915. doi: 10.1155/2015/743915 (PMC4807048; doi:10.1155/2015/743915)

**Supplementary Materials:**

**Figure 1:** a chromatogram showing VCR peak in rat plasma 5.5h after 0.1 mg/Kg VCR iv dose (6h post oral dose of 40 mg/Kg PSZ) measured at 220 nm.

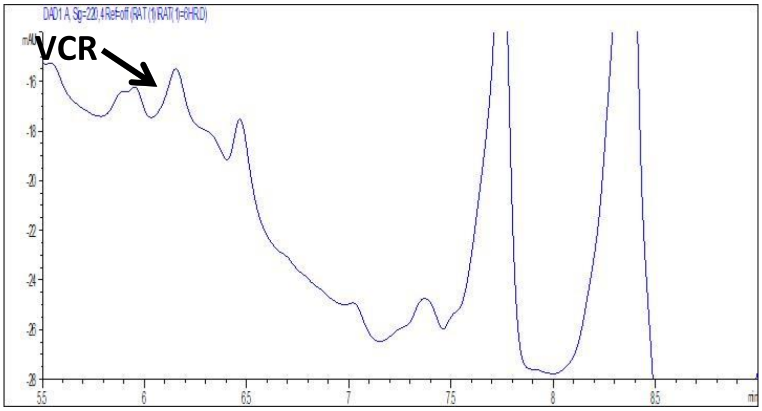


**Figure 2:** A chromatogram showing VCR, PSZ and ITZ in rat plasma 5 minutes after 0.1 mg/Kg VCR iv dose (30 minutes post oral dose of 40 mg/Kg PSZ) measured at 220 nm.

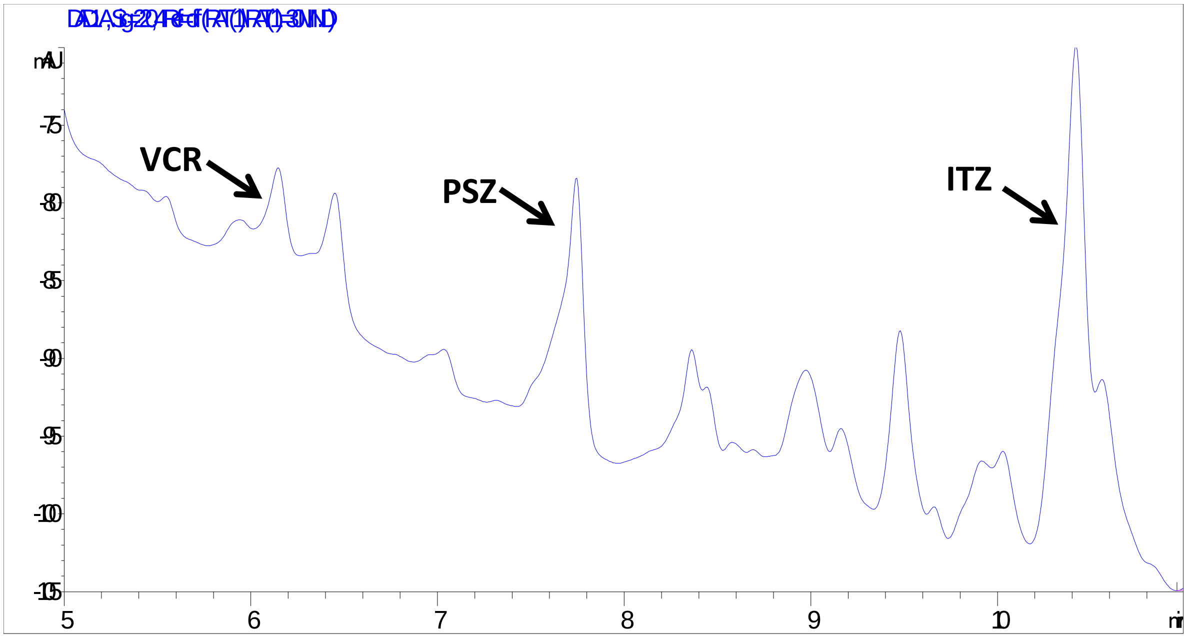

Supplement: Supplementary file 1 — Posaconazole and itraconazole were measured at 262 nm while vincristine showed better sensitivity when measured at 220 nm. The figures below illustrate the vincristine, posaconazole and itraconazole peaks when measured at 220 nm. [file 743915.f1.pdf]
